# Fluorescence Image-Guided Surgery for Thyroid Cancer: Utility for Preventing Hypoparathyroidism

**DOI:** 10.3390/cancers13153792

**Published:** 2021-07-28

**Authors:** Marco Stefano Demarchi, Barbara Seeliger, Jean-Christophe Lifante, Pier Francesco Alesina, Frédéric Triponez

**Affiliations:** 1 Department of Thoracic and Endocrine Surgery and Faculty of Medicine, University Hospitals of Geneva, 4 Rue Gabrielle Perret-Gentil, 1211 Geneva, Switzerland; marcostefano.demarchi@hcuge.ch; 2 Department of Endocrine Surgery, Lyon Sud University Hospitals, 69310 Pierre Benite, France; jean-christophe.lifante@chu-lyon.fr; 3 IHU—Strasbourg, Institute of Image-Guided Surgery, 67091 Strasbourg CEDEX, France; barbara.seeliger@ihu-strasbourg.eu; 4 IRCAD, Research Institute against Digestive Cancer, 67091 Strasbourg CEDEX, France; 5 Department of General, Digestive, and Endocrine Surgery, Strasbourg University Hospitals, 67091 Strasbourg CEDEX, France; 6 Department of Surgery and Center of Minimally Invasive Surgery, Evangelische Kliniken Essen-Mitte, Academic Teaching Hospital of the University of Duisburg-Essen, 45136 Essen, Germany; pieroalesina@yahoo.it; 7 Health Services and Performance Research Lab (EA 7425 HESPER), Université Claude Bernard Lyon 1, 69622 Lyon, France; 8 Department of Surgery, Gemelli Molise Hospital, Università Cattolica del Sacro Cuore, 86100 Campobasso, Italy

**Keywords:** thyroid surgery, thyroid cancer, hypoparathyroidism, fluorescence-guided surgery, near-infrared autofluorescence

## Abstract

**Simple Summary:**

The most frequent post-operative complication in thyroid surgery is hypoparathyroidism leading to temporary or definitive low blood calcium levels. This complication can result from intentional or inadvertent extirpation, trauma, or devascularization of the parathyroid glands. They are located right next to the thyroid, and are responsible for the blood calcium level regulation. Hypoparathyroidism is even more common when a lymph node dissection is needed in addition to thyroidectomy in case of thyroid cancer. The safeguarding of all four parathyroid glands with their vascularization can be extremely challenging, even for experienced surgeons. Fluorescence imaging is a relatively novel intraoperative tool to help identify, visualize, and preserve the parathyroid glands during thyroid surgery. In this review, we summarize the current scientific landscape and the potential benefits of fluorescence imaging to preserve the parathyroid glands and to prevent post-operative hypoparathyroidism in thyroid cancer surgery.

**Abstract:**

**Background:** Hypoparathyroidism is one of the most frequent complications of thyroid surgery, especially when associated with lymph node dissection in cases of thyroid cancer. Fluorescence-guided surgery is an emerging tool that appears to help reduce the rate of this complication. The present review aims to highlight the utility of fluorescence imaging in preserving parathyroid glands during thyroid cancer surgery. **Methods:** We performed a systematic review of the literature according to PRISMA guidelines to identify published studies on fluorescence-guided thyroid surgery with a particular focus on thyroid cancer. Articles were selected and analyzed per indication and type of surgery, autofluorescence or exogenous dye usage, and outcomes. The Methodological Index for Non-Randomized Studies (MINORS) was used to assess the methodological quality of the included articles. **Results:** Twenty-five studies met the inclusion criteria, with three studies exclusively assessing patients with thyroid cancer. The remaining studies assessed mixed cohorts with thyroid cancer and other thyroid or parathyroid diseases. The majority of the papers support the potential benefit of fluorescence imaging in preserving parathyroid glands in thyroid surgery. **Conclusions:** Fluorescence-guided surgery is useful in the prevention of post-thyroidectomy hypoparathyroidism via enhanced early identification, visualization, and preservation of the parathyroid glands. These aspects are notably beneficial in cases of associated lymphadenectomy for thyroid cancer.

## 1. Introduction

The global incidence of thyroid cancer has continuously increased over the past decades [1]. This upward trend has been attributed to improved diagnostic acumen owing to the availability of advanced technology, as well as to lifestyle and environmental changes. Thyroid cancer is currently the most common endocrine cancer worldwide [2,3]. Despite increases in incidence, mortality from thyroid cancer has remained stable over the past decades [1]. The life expectancy of many thyroid cancer patients, especially those younger than 45 years, is not reduced when compared with that of the general population [4]. The prevention of treatment-related morbidity is essential, including minimization of postoperative hypoparathyroidism rates, particularly for young patients with an overall excellent long-term prognosis. The majority of the thyroid cancer burden affects women [3], and the prevention of complications with repercussions on pregnancies is crucial for those of reproductive age.

The treatment of choice for thyroid cancer is surgical resection. Depending on the tumor extent, a total or near-total thyroidectomy with therapeutic or prophylactic lymph node dissection is indicated. Despite the significant benefits of total resection, including increased survival and reduced recurrence rates, extensive resections are typically accompanied by substantially increased complication rates [5]. Thus, the risks and benefits of thyroid cancer surgery must be balanced, targeting complete excision of malignant lesions while preserving critical structures such as the laryngeal nerves and parathyroid glands (PGs), particularly in thyroidectomy with lymphadenectomy. As these challenges are continuously encountered in thyroid cancer surgery, surgical innovations are targeted at minimizing procedure-related morbidity while maintaining therapeutic benefits. 

One such innovation is intraoperative neuromonitoring (IONM) of the recurrent laryngeal nerve (RLN). Over the last two decades, IONM has gained popularity and has become the gold standard for thyroid surgeries in order to prevent injury to the RLN [6], despite initial controversial discussions regarding its benefit [7]. Another emerging technology is fluorescence image-guided surgery (FIGS), a real-time navigation modality based on optical imaging. The use of fluorescence imaging is relatively new in surgery, although it is a widely known method used in biomedical sciences for visualizing cells and tissues in vitro and in vivo [8]. The intraoperative use of light sources in the near-infrared (NIR) excitation range induces the emission of a fluorescent signal via a wavelength shift during tissue interaction [9]. 

In more detail, fluorescence is based on the property of certain substances to absorb external light at a given excitation wavelength and subsequently emit light at a different, longer wavelength with lower energy [10]. As a result, fluorescence imaging involves a sequence in which the tissue of interest is illuminated by a filtered light source at a specific excitation wavelength, light is absorbed by the target tissue, a longer wavelength is emitted, and the fluorescent band is detected by a specifically designed camera [11,12]. The advantage of light in the NIR range is a deeper tissue penetration (<1 cm, with a limited depth resolution beyond 5 mm) [13,14]; thus, NIR imaging devices enable the surgeon to see behind the tissue surface [15]. Other wavelengths (ultraviolet: 200–400 nm or visible: 400–650 nm) are limited for in vivo real-time imaging due to low light penetration into the tissues and autofluorescence from endogenous fluorophores in the body [16,17]. Current in vivo fluorescence imaging systems are thus optimized for the NIR range. This imaging modality provides the surgeon with real-time feedback during the surgical procedure to identify and differentiate surgical targets from normal tissue [12]. Two different modalities of detection of near-infrared autofluorescence (NIRAF) are actually used: a probe-based modality such as the PTeye (by AiBiomed, Santa Barbara, CA, USA) that provides a quantitative, and auditory feedback when the handheld fiber probe touches parathyroid tissue, and the imaging-based modality that uses an NIR light source in conjunction with a filtered camera to identify the autofluorescence of the tissue on a display monitor such as the Fluobeam-800/Fluobeam-LX (Fluoptics, France), PDE Neo II (Hamamatsu, Shizuoka Pref. Japan), and EleVision™ IR Platform (Medtronic, Minnesota, MN, USA), among others.

FIGS is gaining popularity and is being increasingly used in endocrine surgery, including adrenal [15,18], parathyroid, and thyroid surgery [19,20]. With the integration of specific contrast agents and advanced imaging systems, fluorescence guidance has the ability to bring about significant positive change in surgery, leading to better outcomes by improving the visualization of tissues for resection, such as tumors, or those to be preserved, such as nerves, blood vessels, and neighboring organs [12,21]. 

Two fluorescence imaging approaches are important in thyroid and parathyroid surgery, namely, contrast-enhanced fluorescence and autofluorescence imaging. The first technique is based on either intravascular or local administration of an exogenous contrast agent, whereas the second technique uses the intrinsic florescent properties of the target tissue via specific optical modalities [10,22].

### 1.1. Contrast-Enhanced Fluorescence and Autofluorescence

Exogenous contrast-enhanced fluorescence can be induced via the use of fluorescent dyes. For intraoperative imaging, indocyanine green (ICG), a sterile tricarbocyanine dye, is the most commonly utilized fluorophore. ICG absorbs light at excitation wavelengths of 790–805 nm and re-emits light at 835 nm [23]. Other NIR fluorescent agents, such as methylene blue, are used less frequently than ICG due to their potential toxicity [24]. 

In contrast, autofluorescence is based on intrinsic biomolecules that act as endogenous fluorophores. Light emission occurs in the ultraviolet, visible, or NIR spectral range when biological molecules are excited with light at an appropriate specific wavelength [25]. 

Intrinsic tissue fluorescence can be perceived as a background disturbance signal when cells and tissues are labeled with exogenous fluorophores. However, tissue autofluorescence alone has shown great promise when used for research and diagnostic purposes [25]. In particular, the PGs are characterized by exceptional autofluorescence in the NIR range, as discovered by tissue spectral analysis [26,27]. A research group at Vanderbilt University identified the ideal spectral range, with excitation light at 785 nm producing maximal autofluorescence from thyroid and parathyroid tissue within the NIR range (820 nm), with the parathyroid signal ranging from 1.2 to 25 times higher than thyroid, and all surrounding tissues [28] with brown fat and lymph nodes that are known false NIRAF positives in non-parathyroid tissues [13]. This group further explored real-time intraoperative autofluorescence imaging of the PGs [27,29]. The specific molecule acting as an intrinsic fluorophore has not yet been identified. 

Multiple NIR imaging systems are commercially available for clinical use or for research purposes. Some of these devices are optimized for pure autofluorescence imaging, whereas others enable both autofluorescence and ICG contrast-enhanced fluorescence imaging. All autofluorescence devices can detect ICG, but the reverse is usually not true [29]. As the field of potential applications is expanding, customizable systems with a choice of different wavelengths and multispectral fluorescence imagers are being explored [30]. For example, hyperspectral imaging showed distinct spectral signatures of the thyroid glands and PGs compared with the surrounding tissue during neck exploration in a patient [12]. 

Postoperative complications after thyroid surgery may include temporary or permanent hypoparathyroidism, recurrent laryngeal nerve injury or palsy, chyle fistula, Horner’s syndrome, injury to motor nerves in the neck, and hematoma or seroma formation [5,31]. Hypoparathyroidism is the most common complication after total or near-total thyroidectomy due to the accidental resection or devascularization of PGs. Fluorescence imaging has the potential to revolutionize thyroid surgery by significantly reducing hypoparathyroidism, especially for thyroid cancer necessitating an enlarged dissection.

### 1.2. Postoperative Hypoparathyroidism

Transient hypoparathyroidism has been reported in up to 20% of patients undergoing total thyroidectomy for thyroid cancer, with permanent hypoparathyroidism reported in up to 3% [32]. Transient hypoparathyroidism has been reported in up to 26.2% for cases of medullary thyroid carcinoma, with a clear association existing between the extent of lymph node dissection and the observation of four PGs [33]. A systematic review and meta-analysis on bilateral thyroid surgery demonstrated an even higher median rate of transient (27%, interquartile range—IQR: 19–38%) and permanent (1%, interquartile range—IQR: 0–3%) hypocalcemia [34]. The rates increase with the extent of surgery, with symptomatic hypoparathyroidism complications arising in 28.7% of thyroid cancer surgeries (28.4% temporary, 0.3% permanent), as reported from a single center [5]. The permanent hypoparathyroidism rate is reported as 0.4–13.8% [31] and can reach 37% in bilateral neck dissection for thyroid cancer [35].

Although the vast majority of postoperative hypoparathyroidism cases are transient and resolve within 6 months of surgery, a small percentage of patients require calcium and vitamin D supplementation for the rest of their lives. This situation is considered serious and negatively affects quality of life [5]. Women are significantly more likely than men to require permanent calcium replacement for postoperative hypoparathyroidism [36]. Consequently, women of child-bearing age require close monitoring during pregnancy, as they are at risk of hyper- and hypocalcemia. As both conditions have consequences to the fetus and the mother, calcium dose adjustments are frequent [37]. Other implications of postoperative hypoparathyroidism include the economic costs of prolonged hospital stay, additional investigations, procurement of medications, the medical burden associated with lifetime medication, and routine hospital visits for follow-ups. Moreover, there are considerable disease burdens related to chronic kidney failure, increased psychiatric complaints, and basal ganglia calcification, among other sequelae, and possible increases in mortality [38,39].

Surgical techniques that improve the preservation of the PGs and their blood supply are greatly needed. The present review highlights the utility of one such innovation, i.e., fluorescence imaging, in preserving the PGs during total or near-total thyroid resection ± lymph node dissection in the case of thyroid cancer.

The purpose of this review is to provide evidence on the utility of fluorescence-guided surgery for preserving the PGs and preventing hypoparathyroidism, with a particular focus on thyroid cancer surgery. The specific objectives of this review are (A) to determine the utility of the different fluorescence imaging techniques in preventing hypoparathyroidism as a complication of thyroid surgery; (B) to determine the feasibility of FIGS in thyroid cancer surgery, with an emphasis on ease of use, added surgical time, and complications related to the technique; and (C) to identify future directions in the use of fluorescence imaging to prevent postoperative hypoparathyroidism. 

## 2. Materials and Methods

### 2.1. Search Strategy

This systematic literature review was conducted according to the Preferred Reporting in Systematic Review and Meta-Analysis (PRISMA) guidelines [40]. MEDLINE (PubMed), Science Direct, Google Scholar, Medline, Oxford Academic journals, and Cochrane library databases were searched, with MeSH terms and free-text key words used for studies investigating the role of fluorescence-guided surgery in preventing hypoparathyroidism as a complication of thyroidectomy for thyroid cancer. The following search string was then developed: (Fluorescence-guided surgery OR Fluorescence imaging OR Indocyanine green OR Near-infrared imaging OR Autofluorescence) AND (Thyroid cancer OR Thyroid malignancy) AND Thyroidectomy AND (Parathyroid glands OR Hypoparathyroidism). To complete the search, the references within the selected articles were searched as well. The search was conducted until May 2021 by the authors. No age or time limitations were used. After the titles and abstracts had been screened, full-text reports were assessed for eligibility, and references were screened within the selected articles.

A standard form for extracting the following data was used, addressing the characteristics of the selected studies (design, method of randomization), participants (baseline characteristics, tumor type, indication for surgery), and intervention (type of surgery, operative technique, lymph node dissection, type of fluorescence, type of fluorescent dye, dose, timing of administration during surgery, fluorescence system). Outcomes were influence on procedure duration, intra- and postoperative complications, and, particularly, presence of temporary and/or permanent hypoparathyroidism. These data were analyzed and are reported in tables and text. Outcome variables are reported as the absolute number and percentage, for all studies combined and separately for those studies with a control group.

### 2.2. Eligibility Criteria

The studies included in this review were original articles, written in English, published between 2000 and 2021, and reporting on the use of FIGS in preserving the PGs and their blood supply or preventing hypoparathyroidism during thyroid cancer surgery. Articles that report on the use of fluorescence imaging for other procedures or diseases other than thyroid cancer were excluded. Studies not presenting original patient data, animal studies, case reports, conference abstracts, technical notes, and articles written in languages other than English were also excluded.

### 2.3. Methodological Quality Assessment

The Methodological Index for Non-Randomized Studies (MINORS) was used to assess the methodological quality of the included articles [41]. MINORS is a valid instrument designed to assess the methodological quality of non-randomized surgical studies and is based on eight items for noncomparative studies and 12 items for studies with a control group.

## 3. Results

### 3.1. Data Extraction

**Figure 1:**
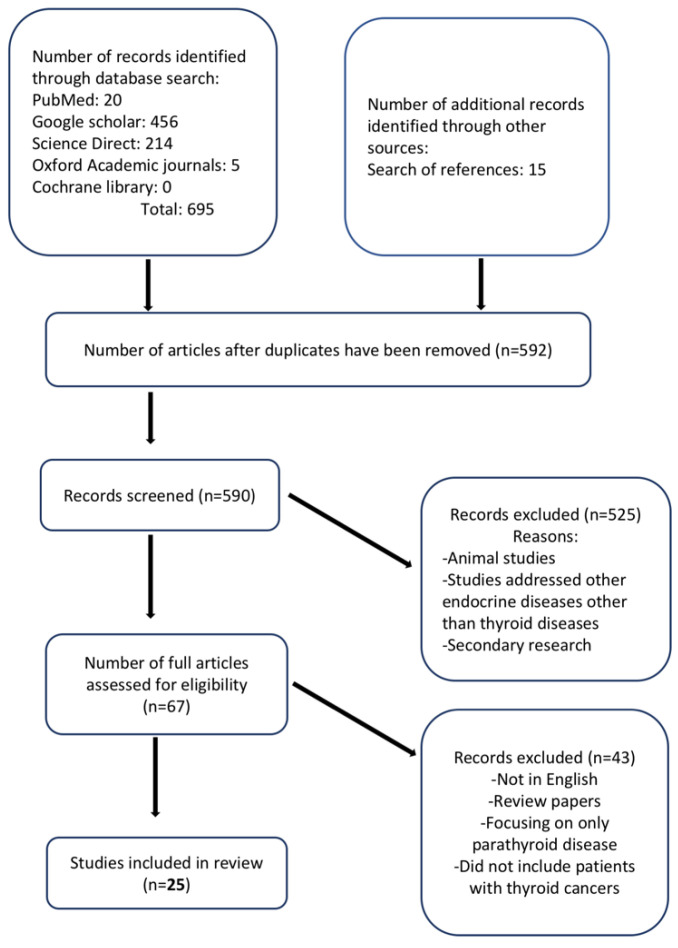
Flow diagram of study selection.

The process of article selection is illustrated in Figure 1. The database searches retrieved 695 articles, and a reference list search retrieved 15 additional studies. After deduplication, 590 articles were screened, of which 67 full articles were retrieved. A total of 25 studies met the inclusion criteria. Overall, the outcomes of nearly 3000 patients were described. All included studies were published between 2016 and 2021, and the mean ages of the included participants ranged from 39.2 to 61.6 years for 22 studies, while three studies did not report the ages of their participants. Two studies reported age ranges (32–70 and 34–73 years, respectively) [42,43]. Five studies were retrospective, and seven studies had a control group. Three studies exclusively evaluated patients with thyroid cancer, whereas the remaining studies assessed patients with thyroid cancer and other thyroid or parathyroid diseases. The study characteristics of the included studies are presented in Table 1.

### 3.2. Methodological Quality of Included Studies

The risk of bias among the included studies was assessed using MINORS, as most of the studies were nonrandomized clinical studies. The maximum possible score was 16 for those without a control group and 24 for those with a control group. The mean quality score was 10.06 (range: 8–14) for noncomparative studies and 20 (range: 18–22) for comparative studies (Table 2). All but one study [53] clearly stated their aim. Nine studies adequately described the follow-up period, including the percentage lost to follow-up. No study included a prospective sample size calculation. All comparative studies had adequate control groups, and the study groups were contemporary with baseline equivalence of groups and adequate statistical analyses. In one study [49], the control group consisted of historical patients.

### 3.3. Type of Fluorescence (Exogenous or Autofluorescence)

**Figure 2:**
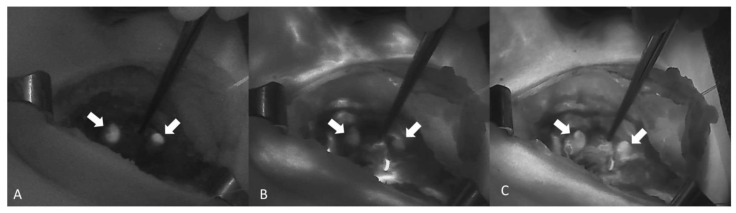
A sequence of images showing ICGA of two PGs (arrows) from A to C. (**A**) Autofluorescence of the PG before the injection of ICG. (**B**,**C**) Diffusion of the ICG contrast agent confirming a well-vascularized PG (Fluobeam LX^®^—Fluoptics©, Grenoble, France).

NIR autofluorescence (NIRAF) alone was used in 11 studies, whereas an exogenous fluorophore was employed in 14 studies. Lerchenberger et al. [54] compared the usefulness of autofluorescence with an exogenous fluorophore, while Ladurner et al. assessed the utility of both autofluorescence and exogenous fluorophore, although not comparatively [59]. Dip et al. [62] compared the use of white light alone with the use of both autofluorescence and white light in the identification of PGs. ICG was used in all but one of the studies using exogenous fluorophores. Enny et al. used 500 mg of fluorescein dye to produce fluorescence in the PGs [47]. The ICG dose was 5 mg in five studies and 2.5 mg in two studies. Two studies reported the administration of repeat doses to a maximum of 5 mg/kg/day [60,61]. The fluorescence system varied from study to study, with the Fluobeam 800 system (Fluoptics, Grenoble, France) being the most common (five studies). The system was not specified in two studies [48,56] (Table 3). An image showing the autofluorescence of the PGs (Figure 2A) and the sequence of ICG angiography (ICGA) (Figure 2B,C) is provided in Figure 2.

### 3.4. Parathyroid Gland Visualization and Preservation

Twenty-three studies reported on the visualization and/or preservation of the PGs during thyroid surgery. Four of these studies [45,49,51,53] demonstrated improved visualization of the PGs using fluorescence imaging compared with conventional thyroid surgery. However, van den Bos et al. [23] showed better visualization with white light compared with NIR imaging. Lerchenberger et al. [54] reported that although autofluorescence demonstrated slightly better visualization of the PGs, it could not indicate whether the blood supply to the gland was still viable, in contrast to the use of ICG. Several studies also reported that fluorescence was used to identify PGs in resected segments or those whose blood supply had been disrupted, which were autotransplanted [57,60,62,67]. In the larger series of Kim et al. (542 patients), no statistical difference in PG visualization was observed between an NIRAF group and the control group (3.91 ± 0.36 vs. 3.90 ± 0.39; *p* = 0.351) [66]. Enny et al. [47] reported better visualization of the PGs using the naked eye than using fluorescein dye, although the difference was not significant. Corresponding data are shown in Table 4.

### 3.5. Postoperative Serum Parathyroid Hormone

Thirteen of the 25 studies reported on postoperative parathyroid hormone (PTH) levels. Some studies using ICG reported that having at least one well-perfused PG (with an ICG score of 2) after thyroid gland resection predicted normal postoperative PTH [44,60,65]. None of the comparative studies found a statistically significant relationship for postoperative PTH levels between study and control groups, except for [66], which found a statistically significant lower incidence of transient hypoparathyroidism in the NIRAF group (33.7% vs. 46.6%; *p* = 0.002). Three studies [45,48,49] demonstrated better PTH levels for fluorescence-guided surgery (Table 4). 

### 3.6. Effects on the Autotransplantation Rate

DiMarco et al. [68] stated that NIRAF imaging might detect inadvertent parathyroidectomy and allow autotransplantation, even though no difference between the NIRAF imaging and control groups was found. Similarly, Ladurner et al. [58] stated that NIRAF imaging assisted in identifying several inferior parathyroid glands that otherwise would have been lost for autotransplantation. Bellier et al. [69] found that NIRAF imaging can help detect the accidental removal of parathyroid glands and that 60% of these glands can be spared and autotransplanted during the surgery. In the controlled study of Benmiloud et al. [70], NIRAF imaging appeared to reduce the autotransplantation rate (from 15% to 2.1%) and the inadvertent parathyroid resection rate (from 7.2% to 1.1%) thanks to improved parathyroid gland identification. These findings were confirmed later by the same author [45] in a study utilizing NIRAF imaging to detect a reduction in the inadvertent resection rate (from 11.7 to 2.5; *p* = 0.006) and the autotransplantation rate (from 13.3 to 3.3; *p* = 0.009).

Kim et al. [64] found that the rate of incidental parathyroidectomy was higher in the conventional (14%) versus NIRAF imaging group (6%) (*p* = 0.039) despite similar autotransplantation rates (4% vs 6%, respectively; *p* = 0.562). This was in line with another study [66] reporting that the number of inadvertently resected PGs (in the pathologic specimen) was significantly lower in the NIRAF group (12.8% vs 6.9%; *p* = 0.021), but that the number of autotransplanted PGs in both groups was similar.

The autotransplantation rate based on ICG angiography was approximately 17% [52]; however, some authors [49] found it significantly increased not only in the ICG group compared to the control group (36% vs. 12%; *p* = 0.0001), but also comparing the ICG group with the NIRAF group [54]. 

Several authors [49,71] have stated that ICG angiography can guide more appropriate autotransplantation without compromising postoperative parathyroid function. This is in contrast with Razavi et al. [48], who assert that ICG angiography may lead to unnecessary parathyroid autotransplantation because low-flow ICG patterns are not associated with postoperative PTH changes or transient hypocalcemia.

### 3.7. Postoperative Serum Calcium

As shown in Table 4, 17 studies reported on postoperative hypocalcemia among their patients. Some demonstrated an absence of postoperative hypocalcemia [44,60,66].

Benmiloud et al. reported a significantly lower rate of hypocalcemia in the NIRAF group compared with the standard group (14.3% vs. 21.7%; *p* = 0.007) [45]. Permanent hypocalcemia was reported in only one study, where three patients in the control group (conventional surgery) required calcium supplementation over 6 months, as opposed to no patients in the study group (autofluorescence) [53]. Razavi et al. [48] observed hypocalcemia in 7.9% of the ICG group versus 3.9% of the conventional group. Dip et al. [62] reported higher mean serum calcium levels in the study group compared with the control group, although this finding did not reach a level of statistical significance. In a study in which patients with an ICG score of at least 2 in one PG were randomized into two groups, of which one group received calcium supplementation and the other did not [65], none of the participants in either group had hypocalcemia. Moreover, there were no significant differences in serum calcium levels between the two groups.

### 3.8. Additional Duration of Surgery

The duration of surgery or additional time spent on NIR imaging during surgery was given in 10 of the 25 studies, with an additional time of 3–10 min for the procedure. In one study [45], the procedures in the NIRAF group required an additional time of 8 min compared with those in the standard group, which is in contrast with another study [64]. The total operating time was not significantly affected by NIRAF imaging in another study [57]. In contrast, a different study reported that NIRAF added 5–8 min to the operating time, with ICG use adding another 3 min [54]. Four other studies [57,59,60,62] also reported that NIR imaging required an additional time of 3–10 min. 

The average durations of the entire procedure were 92 ± 32 min and 109.1 ± 49.8 min in two studies [50,52], with 5 min spent on NIR imaging in [50].

### 3.9. Complications Related to Technique

No complications attributed to the technique were reported in any of the articles.

### 3.10. Cost

None of the included studies described the cost implication of using NIR imaging.

## 4. Discussion

An increasing number of scientific reports have investigated the utility of fluorescence-guided surgery in preserving the PGs and preventing hypoparathyroidism as a complication of thyroid surgery. However, only three studies have addressed thyroid cancer patients alone. In contrast, in the majority of reports, the patient cohorts are mixed, including both benign and malignant indications and thyroid as well as parathyroid diseases. According to the available literature, NIRAF imaging improves the visualization and preservation of the PGs during thyroidectomy. This technique is feasible and safe, as NIRAF imaging is non-invasive. Moreover, no NIR imaging-related complications were reported in any of the studies, including the studies with ICG injection.

Among the included reports, NIRAF was used in 11 studies, and an exogenous fluorophore was employed in 14 studies. Because NIRAF exploits the endogenous fluorophore of the PGs, no additional time is required to administer an exogenous dye [42]. Consequently, the only equipment required for NIRAF imaging is a fluorescence camera system. A comparative study of NIRAF and ICG imaging found only a minimal difference in the usefulness of both techniques for identifying the PGs [54]. However, preserved perfusion can be visualized only by the introduction of a contrast agent (e.g., ICG) via the blood stream. Therefore, NIRAF is technically inappropriate for assessing the integrity of arterial supply and venous outflow. In contrast to ICG, NIRAF cannot determine the vascular integrity of the PG. ICG has a short half-life of 3–5 min and is eliminated by the hepatic system after approximately 15 min [19]. Hence, repeated doses can be required and safely administered [50,60,61]. The toxic dose in adults is 5 mg/kg circulating per time point [19]. All doses administered in the included studies were well below this threshold. ICG was administered intravenously in all cases, and a duration of 1–3 min was required for the PG to take up the ICG [44,48,52,72]. Upon excitation with NIR light, the fluorescence produced by the gland was scored as 0, 1, or 2 if the gland appeared black (not vascular), grey (partially vascular), or white (vascular), respectively [48,60,61]. 

The prevailing causes of hypoparathyroidism after thyroidectomy are disruption of the blood supply to the glands and their incidental removal. These complications are more frequently seen in thyroidectomy for thyroid cancer [19], especially with accompanying lymph node resection. Because of the ability of NIR imaging to visualize the PGs and to detect impaired blood supply to the glands during thyroid cancer surgery, this technique is invaluable in averting hypoparathyroidism. In a randomized controlled trial, there was a significant difference between the number of PGs identified with NIRAF compared with conventional surgery. The percentage of patients with four identified PGs was higher in the NIRAF group than in the standard-care group (47.1% vs. 19.2%, respectively; *p* < 0.001). Consequently, the number of incidentally resected PGs was significantly lower in the NIRAF group [45]. In another controlled study, visual and ICGA assessment of vascularity showed agreement in 87% of cases. ICGA prevented autotransplantation of 19 (6.8%) glands that were shown to be adequately perfused on ICGA, but would have been transplanted if visual inspection alone had been used. Interestingly, autotransplantation of the PGs was significantly more common among the ICGA group than the control group (36% vs. 12%, *p* = 0.0001) [49].

In a retrospective study [51], the use of white light and the use of ICG and NIR imaging were compared for identifying the PGs at an early dissection stage. The surgeons documented the number of PGs that were visualized with the naked eye using white light. Next, the operating room’s light was turned off, and the surgical field was illuminated with NIR light. The number of PGs visualized with NIR light was significantly higher than in white light. Furthermore, the integrity of the blood supply to the PGs was assessed via ICGA in cases of ambiguity. Perfusion was intact in all cases; hence, no autotransplantation was performed. While the majority of the studies in this review reported improved visualization of the PGs in favor of NIR fluorescence imaging, one study [50] reported slightly better visualization of the glands in white light. It is possible that the bright background signal of highly vascularized malignant tissue may have interfered with visualization on fluorescence imaging. In the same study, the majority of surgeons considered NIR fluorescence imaging to be advantageous. The reduction in both the autotransplantation rate and the inadvertent parathyroid resection rate, which are both risk factors for postoperative hypocalcemia, reported by studies in this review is corroborated by Fanaropoulou et al. [19].

The largest series reported in the literature included 542 patients [66] and focused exclusively on thyroid cancer. This study found no significant differences in the number of visualized PGs between the NIRAF group and the conventional group. This finding could be due to the substantial experience of the surgeon. The same article still reported a significantly lower incidence of transient hypoparathyroidism in the NIRAF group compared with the control group. Based on the large series included in this study and the quality of the study, this statement seems to be well supported. 

Visualization of the PGs alone may not be sufficient to prevent postoperative sequelae. Dissection may lead to inadvertent damage to the end arteries that supply the PGs. Hence, some studies addressed the assessment of PGs after resection of the thyroid gland to ensure its viability and/or the need for autotransplantation [47,48,52]. Enny et al. [47] found that the identification of PG viability by NIR imaging visualization of intact vascularity at the end of the procedure is associated with a reduced need for routine postoperative calcium and vitamin D supplementation, along with reductions in accompanying costs and duration of hospital stay. However, some authors [48] recommend that surgeons interpret these results with caution, as their study on the use of ICGA did not show a significant improvement in thyroid surgery outcomes. In particular, poor vascular perfusion on ICGA did not correlate with a postoperative reduction in PTH levels or transient hypocalcemia. The ability of ICGA to detect impaired perfusion and to induce a change in surgical strategy was demonstrated in colorectal surgery with a positive outcome [23].

Thirteen studies reported on postoperative PTH levels, whereas 17 studies reported serum calcium levels. Jin et al. [73] performed a small study assessing PG perfusion via ICGA after resection of the first thyroid lobe, with serum PTH and calcium levels assayed on postoperative day (POD) 1, 7, and 14, and 6 months after operation. PGs that had been accidentally devascularized during the surgery were identified using ICGA and were autotransplanted. Their findings showed that the ICGA score was predictive of postoperative PTH levels, as all of the 22 patients with at least one PG having an ICG score of 2 (84.6%) had normal postoperative PTH levels, while half of those with poor vascularization on ICGA developed transient hypoparathyroidism. Similar findings were reported in another study [60], where all patients with at least one well-vascularized gland as detected by ICGA (83.3%) had normal serum PTH levels. One of these patients had asymptomatic hypocalcemia at POD 10. In contrast, two out of six patients with poor vascularization on ICGA developed transient hypoparathyroidism. Overall, postoperative supplementation with calcium or vitamin D was averted in all patients. Thus, ICGA can be used intraoperatively to determine whether at least one PG remains vascularized and functional, in order to predict the absence of postoperative hypoparathyroidism.

The use of fluorescein dye to visualize the PGs was found to be superior to naked eye assessment with regard to preventing hypocalcemia. Among patients with >3 PGs visualized via fluorescein dye, none suffered clinical hypocalcemia, as opposed to more than 20% with clinical hypocalcemia when >3 PGs were visualized with the naked eye only [47]. 

Several comparative studies [45,53,64] reported better postoperative PTH and calcium levels among NIR imaging groups compared with conventional surgery groups, but the opposite trend was observed by others [48,49]. Among the latter, Razavi et al. suggested that intraoperative monitoring of PTH levels should remain, which they suggest as a better method than ICGA for predicting PG vascularization. Rudin et al. opined that the potential of ICGA to quickly identify PGs and to positively alter surgical strategy cannot be ignored, but that more research should be conducted to determine whether this practice reduces the risk of postoperative hypoparathyroidism.

This controversy is fueled by a lack of standardization among NIR fluorescence techniques, such as in the timing of assessment and the quantification of fluorescent signal intensity. PG visualization can be enhanced with NIR imaging prior to any dissection or during thyroid dissection. Early identification can prevent inadvertent resection or impairment of vascular supply. In this way, NIR imaging can contribute to the preservation of all PGs. The distinction between the more intense fluorescence of the PGs compared with that of the lymph nodes can provide guidance during lymphadenectomy. Late detection, such as that performed on the specimen, can allow functional recovery of the PG with autotransplantation. Contrast-enhanced fluorescence imaging with intravenous ICG or fluorescein injection can visualize preserved versus impaired perfusion (e.g., congruent results shown intraoperatively for adrenal perfusion) [9]. However, except for quantitative contrast-enhanced NIR imaging research protocols [74], fluorescent signals and ICG scores are generally assessed subjectively. For ICG injections, the rate at which the fluorescence signal intensity increases, as well as the added signal intensity produced by reinjections, can only be objectively assessed with a quantitative approach, which has not yet been integrated into commercially available NIR imaging systems. 

Furthermore, it can be concluded from this review that the use of NIR imaging is safe and feasible. No study reported any complication arising from this technique. Rare allergic reactions to ICG have been previously reported [75]. These reactions are particularly attributable to iodine or sodium iodide, which is often added to improve fluorophore solubility [19]. Therefore, patients with iodine allergy are not eligible for ICG administration. However, ICG solutions without iodine are commercially available (e.g., INFRACYANINE, SERB S.à.r.l.); thus, this categorical exclusion of patients for iodine allergy or hyperthyroid status might be lifted in the future.

Several studies in this review reported minimal added time (3–10 min) for NIR imaging but this was shown as not statistically significative [45,54,62]; anyway, this added time would be negligible, considering the benefits of the technique. Similar findings were reported by van den Bos et al. [50]. Interestingly, and in contrast to the observation of a longer duration with the use of fluorescence imaging, two studies reported that more time was spent during conventional surgery [45,64]. Although this trend was not elucidated, it may have been due to a more rapid identification of the glands using autofluorescence, without the added duration needed for the administration and uptake of an exogenous fluorophore. 

Concerning ICGA, among the authors who analyzed this factor, the procedure required an additional operative time of 2–5 min; however, this approach has the benefit of eliminating any doubt on the viability of the explored PGs. Moreover, according to some authors [47,61], ICGA can also prevent systematical postoperative calcium supplementation, thus eventually reducing the hospital stay. The use of fluorescence imaging in surgery has been reported as cost-effective [44,76]. However, no study in the present review stated the cost implication of NIR imaging, which is mainly associated with equipment purchase. The camera systems required for NIR imaging can be expensive. However, once a fluorescence system is installed, autofluorescence creates no further cost. Hence, the additional cost is limited to the price of the exogenous fluorophore used per patient [77] and the sterile cover. When assessed in light of the deleterious health effects and economic burden of hypoparathyroidism and hypocalcemia, NIR imaging can be viewed as very cost-effective, as demonstrated by its positive impact. 

In a cost–benefit analysis on the usefulness of routine intraoperative intact parathyroid hormone (IOPTH) assay in parathyroid surgery, it was recommended to reserve the use of IOPTH assays for select cases due to its high cost (EUR 170 for five rapid IOPTH assays vs. EUR 125 for five delayed PTH assays, plus added OR time amounting to EUR 15/min in the authors’ institution) [78].

The NIR fluorescence equipment cost is amortized more rapidly when it is used by several disciplines within a hospital environment, as abovementioned NIRAF use then generates no additional cost, and a 25 mg bottle of ICG costs around USD 80 and could, in appropriate sterile conditions, be used for 3 to 5 patients in the same day.

The majority of the analyzed articles appear to agree that NIRAF imaging allows a better identification of PGs but that its impact on autotransplantation rate is difficult to determine. Notably, some authors state that NIRAF imaging allows the identification and autotransplantation of accidentally resected glands (e.g., found on the specimen or in central neck dissection) and that the better early identification of PGs in an expert surgeon’s hands will be able to spare these PGs from vascular disruption, thereby reducing the need for autotransplantation [45,70]. 

ICG angiography is commonly accepted to assist in decision-making on autotransplantation. However, it appears to lead to an increase in the autotransplantation rate, and some authors have mentioned the risk of unnecessary parathyroid autotransplantation on the basis of low-flow ICG patterns [48]. They suggested limiting autotransplantation only to clearly devascularized glands that cannot be preserved.

Moreover, metastatic lymph nodes from papillary thyroid cancer are known false positives presenting an high autofluorescence pattern similar to the one of PGs [55], and reimplantation of an autofluorescent nodule found in the central neck dissection should be done with caution if not clearly a PG or confirmed with an intraoperative frozen biopsy analysis [79].

### Limitations

The present review has several limitations. First, only three studies addressed thyroid cancer exclusively, and findings in the other studies were not separated according to the indication for surgery. Hence, there are insufficient data to draw any conclusions regarding the impact of fluorescence imaging on the outcome of thyroid cancer surgery in particular. Further studies should include a more detailed investigation of the observations of a potentially increased benefit of NIR imaging for extended oncological surgery, including neck dissection. Second, the majority of the available and included studies were of limited quality, with only three RCTs (randomized controlled trials). The retrospective nature of some studies [48,49,64] hinders correction for confounders between the study and control groups. However, this aspect could enhance the comparability of both groups by allowing the selection of similar controls for each case. Third, the majority of included studies were small studies with a lack of prospective sample size calculation, which may have affected the observed outcomes. 

Another limitation is the variability in NIR imaging use among the reports. In most studies, not all four PGs were visualized. Therefore, the vascularization and viability of the unidentified glands remained unknown, which may have affected the outcome of the surgery. Furthermore, the subjective nature of estimating the fluorescence level in a gland is a limitation, as the available systems only allow qualitative assessments. This limitation leads to subjective interpretation and interobserver differences in scoring the fluorescence intensity of a gland. To eliminate this limitation, future studies should integrate computer-assisted quantitative contrast-enhanced NIR imaging evaluations [12] or pixel/color-analyzing computer programs, as suggested by Fanaropoulou et al. [19]. 

## 5. Conclusions

Fluorescence-guided surgery is useful for preventing post-thyroidectomy hypoparathyroidism and is also feasible and safe. As this review has shown, fluorescence-guided surgery enhances early identification, visualization, and preservation of the PGs and may reduce the incidence of postoperative hypoparathyroidism and hypocalcemia. Moreover, this technique assists in the identification of accidentally resected glands on the specimen for subsequent autotransplantation. In thyroid cancer surgery, such intraoperative guidance is particularly beneficial for extended dissection and lymphadenectomy. Further studies are needed to focus on thyroid cancer surgery, as current data are scarce. Moreover, standard-of-practice guidelines are needed to identify the ideal timepoint(s) for NIRAF and ICG imaging during thyroid cancer surgery to optimize the beneficial influence of NIR imaging and proper adoption of the technique. 

Most authors highlighted the potential of fluorescence imaging to curtail the need for postoperative supplementation with calcium and vitamin D, especially when paired with the surgeon’s critical decision-making skills. However, the articles published thus far have analyzed outcomes for highly experienced/high-volume surgeons. Thus, more high-quality research is required to validate the long-term advantages of these techniques over use of the naked eye, especially in the case of less-experienced surgeons, who will most likely benefit more from NIR imaging techniques.

## Figures and Tables

**Table 1:** Study characteristics of included studies. * Mean ± standard deviation unless otherwise stated.

s/n	Study	Number of Patients	Age (Years) *	Study Design	Indication for Surgery	Type of Surgery
1	(Jin et al., 2018) [44]	26	49.6 ± 14.7	Prospective cohort study	Thyroid cancer (23%) Benign thyroid disease (77%)	Open total thyroidectomy
2	(Benmiloud et al., 2020) [45]	241	53.6 ± 13.6	Prospective multicenter RCT	Thyroid cancer 56 (23.2%) Benign thyroid disease 185 (76.8%)	Open total thyroidectomy
3	(Kose et al., 2020) [46]	310 patients; 173 underwent thyroid surgery	55.6 ± 15.2	Prospective clinical study	Thyroid cancer 39 (13%) Benign thyroid nodule/multinodular goiter 115 (37%) Hyperthyroidism 19 (6%) Hyperparathyroidism 137 (44%)	Open total thyroidectomy 139 (45%) Thyroid lobectomy 34 (11%) Parathyroidectomy 137 (44%)
4	(Enny et al., 2020) [47]	72	39.2 ± 11.9	Prospective cohort study	Thyroid cancer 14 (18%) Benign thyroid disease 58 (69%)	Open total thyroidectomy
5	(Razavi et al., 2019) [48]	111 43—ICG 68—conventional	ICG: 50.51 ± 1.98 Conventional: 51.56 ± 1.46	Retrospective case–control study	**ICG**: Thyroid cancer (36.8%) Benign conditions (45.1%) **Conventional**: Thyroid cancer (34.9%) Benign conditions (44.6%)	Laparoscopic total or completion thyroidectomy
6	(McWade et al., 2019) [43]	30	Range: 32–70	Prospective clinical study	Thyroid diseases including cancer 12 (40%) Parathyroid diseases 15 (50%) Both 3 (10%)	Open thyroidectomy and parathyroidectomy
7	(Rudin et al., 2019) [49]	210 86—ICG 124—conventional	ICG: 47 Control: 49	Retrospective case–control study	**ICG**: Thyroid cancer (56%) Benign conditions (44%) **Control**: Thyroid cancer (65%) Benign conditions (35%)	Open total/near-total thyroidectomy
8	(van den Bos et al., 2019) [50]	30 surgeries in 26 patients	56.3 ± 16	Prospective clinical study	Suspected thyroid cancer 17 (56.7%) Proven thyroid cancer 7 (23.3%) Benign thyroid disease 6 (20.0%)	Open total thyroidectomy, completion thyroidectomy, and hemithyroidectomy
9	(Falco et al., 2017) [51]	74	48.4 ± 13.5	Retrospective clinical study	Thyroid cancer 35 (47%) Goiter 23 (31%) Primary hyperparathyroidism 13 (18%) Hyperthyroidism 3 (4%)	Not stated
10	(Lang et al., 2017) [52]	94	54.5 ± 15.0	Prospective clinical study	Thyroid cancer 12 (17.1%) Benign pathology 38 (54.3%) Graves’ disease/toxic goiter 15 (21.4%) Indeterminate cytology 5 (7.1%)	Open total thyroidectomy
11	(Serra et al., 2020) [53]	105 45—study 65—control	Study group: 61.4 ± 15.5 Control: 61.6 ± 12.1	Prospective case–control study	**Study**: Malignant 20 (33.3%) Benign 40 (66.7%) **Control**: Malignant 8 (17.8%) Benign 37 (82.2%)	Open total thyroidectomy
12	(Lerchenberger et al., 2019) [54]	50	47.2	Prospective clinical study	Thyroid cancer 12 Benign thyroid disease 16 Parathyroidectomy 17	Open total thyroidectomy, hemithyroidectomy, and parathyroidectomy
13	(De Leeuw et al., 2016) [55]	35	40.9	Prospective clinical study	Benign and malignant thyroid diseases	Open total thyroidectomy, hemithyroidectomy, and parathyroidectomy
14	(Llorente et al., 2020) [56]	50	52 ± 12.9	Prospective cohort study	Thyroid cancer (70%) Multinodular goiter (26%) Graves’ disease (4%)	Open total thyroidectomy
15	(S. W. Kim et al., 2016) [42]	8	Range: 34–73	Prospective clinical study	Papillary thyroid cancer	Open total thyroidectomy and hemithyroidectomy
16	(S. W. Kim et al., 2018) [57]	38	Not stated	Prospective clinical study	Papillary thyroid cancer	Open total thyroidectomy (44.7%) Unilateral lobectomy (55.3%)
17	(R. Ladurner et al., 2018) [58]	21	Not stated	Prospective clinical study	Thyroid diseases including thyroid cancer	Open thyroidectomy
18	(Roland Ladurner et al., 2019) [59]	117	49.9 Range: 19–81	Prospective clinical study	Thyroid cancer (21.3%) Thyroid benign (42.7%) Parathyroid disease (35.9%)	Total thyroidectomy, partial thyroidectomy, and parathyroidectomy
19	(Vidal Fortuny et al., 2016) [60]	36	49.8 ± 15.7	Prospective clinical study	Thyroid cancer (22.2%) Benign thyroid diseases (77.8%)	Total thyroidectomy
20	(Vidal Fortuny et al., 2018) [61]	196	Not stated	Prospective RCT	**Study group**: Malignancy 30 (41%) Benign 40 (59%) **Control**: Malignancy 17 (23%) Benign 56 (77%)	Completion thyroidectomy and total thyroidectomy
21	(Dip et al., 2019) [62]	170	47.3 ± 13.6	Prospective RCT	Thyroid cancer (48.2%) Benign conditions (51.8%)	Total thyroidectomy
22	(Zaidi et al., 2016) [63]	27	43.9 ± 1.0	Prospective feasibility study	Thyroid cancer (37.0%) Multinodular (48.2%) Graves’ disease (14.8%)	Total thyroidectomy, completion thyroidectomy, and hemithyroidectomy
23	(Y. S. Kim et al., 2020) [64]	300 100—study 200—control	Study: 51.6 ± 15.2 Control: 50.2 ± 15.5	Retrospective case–control study	Thyroid cancer (55.3%) Benign thyroid disease (44.7%)	Total thyroidectomy
24	(Jin & Cui, 2020) [65]	56 28—test group 28—control group	42.68 ± 11.70	Randomized control trial	**Study group**: Malignancy 9 (32.1%) Benign thyroid disease 19 (67.9%) **Control**: Malignancy 9 (32.1%) Benign thyroid disease 19 (67.9%)	Total thyroidectomy
25	(D. H. Kim et al., 2021) [66]	542 261—NIRAF group 281—control group	NIRAF group: 51.30 ± 12.44 Control group: 52.83 ± 10.92	Retrospective study with historical control	All thyroid cancer patients	Total thyroidectomy with unilateral or bilateral central neck dissection

**Table 2:** Methodological Index for Non-Randomized Studies.

Study	A	B	C	D	E	F	G	H	I	J	K	L	Total
(Jin et al., 2018) [44]	2	2	2	2	2	2	2	0	X	X	X	X	14/16
(Benmiloud et al., 2020) [45]	2	2	2	2	2	2	2	0	2	2	2	2	22/24
(Kose et al., 2020) [46]	2	2	2	2	2	0	0	0	X	X	X	X	10/16
(Enny et al., 2020) [47]	2	2	2	1	2	1	0	0	X	X	X	X	10/16
(Razavi et al., 2019) [48]	2	2	2	2	2	2	2	0	2	2	2	2	22/24
(McWade et al., 2019) [43]	2	2	2	2	2	0	0	0	X	X	X	X	10/16
(Rudin et al., 2019) [49]	2	2	0	2	2	2	2	0	2	0	2	2	18/24
(van den Bos et al., 2019) [50]	2	2	2	2	2	1	0	0	X	X	X	X	11/16
(Falco et al., 2017) [51]	2	2	2	2	1	0	0	0	X	X	X	X	9/16
(Lang et al., 2017) [52]	2	2	2	2	2	0	0	0	X	X	X	X	10/16
(Serra et al., 2020) [53]	1	2	2	2	2	2	0	0	2	2	2	2	19/24
(Lerchenberger et al., 2019) [54]	2	2	2	1	1	0	0	0	X	X	X	X	8/16
(De Leeuw et al., 2016) [55]	2	2	2	2	0	0	0	0	X	X	X	X	8/16
(Llorente et al., 2020) [56]	2	2	2	2	0	2	0	0	X	X	X	X	10/16
(S. W. Kim et al., 2016) [42]	2	2	2	2	1	0	2	0	X	X	X	X	11/16
(S. W. Kim et al., 2018) [57]	2	2	2	2	1	0	2	0	X	X	X	X	11/16
(R. Ladurner et al., 2018) [58]	2	2	2	2	1	0	0	0	X	X	X	X	9/16
(Roland Ladurner et al., 2019) [54]	2	2	2	2	1	0	0	0	X	X	X	X	9/16
(Vidal Fortuny et al., 2016) [60]	2	2	2	2	2	0	0	0	X	X	X	X	10/16
(Vidal Fortuny et al., 2018) [61]	2	2	2	2	2	2	2	0	2	2	2	2	22/24
(Dip et al., 2019) [62]	2	2	2	2	2	2	2	0	2	2	2	2	22/24
(Zaidi et al., 2016) [63]	2	2	2	2	1	0	2	0	X	X	X	X	11/16
(Y. S. Kim et al., 2020) [64]	2	2	1	2	1	2	0	0	2	1	2	2	17/24
(Jin & Cui, 2020) [65]	2	2	2	2	1	2	0	0	2	2	2	2	19/24
(D. H. Kim et al., 2021) [66]	2	2	1	2	2	2	2	0	2	1	2	2	19/24

Items are scored as 0 (not reported), 1 (reported but inadequate), or 2 (reported and adequate). The maximum score is 16 for noncomparative studies and 24 for comparative studies. A—A clearly stated aim; B—Inclusion of consecutive patients; C—Prospective collection of data; D—Endpoints appropriate for the aim of the study; E—Unbiased assessment of study endpoint; F—Follow-up period appropriate for the aim of the study; G—Loss to follow-up less than 5%; H—Prospective calculation of the study size; I—Adequate control group; J—Contemporary groups; K—Baseline equivalence of groups; L—Adequate statistical analyses.

**Table 3:** Fluorescence type, dosage, timing, and fluorescence system.

Study	Autofluorescence or Exogenous Dye	Type of Exogenous Dye	Dose	Timing of Administration	Fluorescence System
(De Leeuw et al., 2016) [55]	AF	NA	NA	NA	Fluobeam 800 clinical system (Fluoptics, Grenoble, France)
(Serra et al., 2020) [53]	AF	NA	NA	NA	Custom NIRAF device (Thorlabs GmbH, Dachau, Deutschland and CCD Sony ICX254AL image detector)
(McWade et al., 2019) [43]	AF	NA	NA	NA	Overlay tissue imaging system (OTIS)
(Benmiloud et al., 2020) [45]	AF	NA	NA	NA	Fluobeam 800 system (Fluoptics)
(Kose et al., 2020) [46]	AF	NA	NA	NA	Fluobeam device (Fluoptics)
(S. W. Kim et al., 2016) [42]	AF	NA	NA	NA	Digital camera, NIR light-emitting diode (LED), and IR illuminating lights
(S. W. Kim et al., 2018) [57]	AF	NA	NA	NA	M780L3-C1, Thorlabs, Newton, NJ, USA and INFRALUX-300, Daekyung Electro Medical Co., Republic of Korea
(R. Ladurner et al., 2018) [58]	AF	NA	NA	NA	NIR/ICG endoscopic system (Karl Storz, Tuttlingen, Germany).
(Y. S. Kim et al., 2020) [64]	AF	NA	NA	NA	Fluobeam; Fluoptics, Grenoble, France
(D. H. Kim et al., 2021) [66]	AF	NA	NA	NA	Modified DSLR camera and LED (M780L3-C1, Thorlabs, New Jersey, USA) light source
(Dip et al., 2019) [62]	White light alone vs. AF + white light	NA	NA	NA	Fluobeam 800 system (Fluoptics)
(Lerchenberger et al., 2019) [54]	AF vs. exogenous	ICG	5 mg	After lateral mobilization of the thyroid and exposure of the RLN	NIR/ICG endoscopic system (Karl Storz, Tuttlingen, Germany).
(Roland Ladurner et al., 2019) [59]	AF and exogenous	ICG-Pulsion	5 mg	After lateral mobilization of the thyroid gland	Storz laparoscopic NIR/ICG imaging system
(Falco et al., 2017) [51]	Exogenous	ICG	0.5 mL	After exposure of the thyroid gland	NIRL (near infrared light) using a laser system
(Lang et al., 2017) [52]	Exogenous	ICG	2.5 mg	After resection of the thyroid gland	SPY fluorescent imaging system (Novadaq Technologies, Inc., Mississauga, ON, Canada)
(Jin et al., 2018) [44]	Exogenous	ICG	5 mg	After adequate exposure of each central neck compartment	Intraoperative navigation system (Digi-MIH-001-I, Digital Precision Medicine Technology Co., Ltd., Beijing, China); fluorescence imaging system
(Llorente et al., 2020) [56]	Exogenous	ICG	Not stated	After thyroid resection	Not specified
(Enny et al., 2020) [47]	Exogenous	Fluorescein dye	500 mg	After thyroid gland resection	LED blue light
(Razavi et al., 2019) [48]	Exogenous	ICG	5 mg	At the end of surgery	Not specified
(Rudin et al., 2019) [49]	Exogenous	ICG	6 mL (3 per side)	At the end of surgery	Laparoscopic PINPOINT camera (NOVADAQ, ON, Canada)
(van den Bos et al., 2019) [50]	Exogenous	ICG	7.5 mg twice, i.e., 15 mg	Before and after resection of the thyroid gland	Laparoscopic fluorescence imaging system (Karl Storz GmbH & Co., Tuttlingen, Germany)
(Vidal Fortuny et al., 2016) [60]	Exogenous	ICG	3 to 5 mL doses (75–150 mg) up to 5 mg/kg/day	After excision of the thyroid gland	Laparoscopic PINPOINT^®^ camera (Novadaq, ON, Canada)
(Vidal Fortuny et al., 2018) [61]	Exogenous	ICG	2.5 mg doses up to 5 mg/kg/day	After excision of the thyroid gland	NIR camera (Pinpoint^®^; Novadaq, Toronto, ON, Canada
(Zaidi et al., 2016) [63]	Exogenous	ICG	5 mg	Before and after thyroid resection	Pinpoint video-assisted NIR system (Novadaq, Inc., Toronto, ON, Canada)
(Jin & Cui, 2020) [65]	Exogenous	ICG	5 mg/kg	After resection of thyroid lobes	Digi-MIH-I-001, Digital Precision Medicine Technology Co., Ltd, Beijing, China

AF—Autofluorescence; ICG—Indocyanine green; NA—not applicable.

**Table 4:** Parathyroid gland visualization and preservation, postoperative serum parathyroid hormone levels, and postoperative serum calcium levels.

Study	PG Visualization and Preservation	Postoperative Serum PTH	Postoperative Serum Calcium
(Jin et al., 2018) [44]	Among 104 PGs, 86 were identified.	In the 22 patients with at least one PG with an ICG score of 2, postoperative PTH levels were normal. In four patients, ICG did not demonstrate a well-vascularized PG. Two of these patients developed transient hypoparathyroidism.	None of the patients developed hypocalcemia at the time of measurement.
(Benmiloud et al., 2020) [45]	The rate of patients with four identified PGs was higher in the NIRAF group (47.1% (95% CI, 38.5–56.4%)) than in the standard-care group (19.2% (95% CI, 12.1–26.2%); *p* < 0.001)	The PTH concentration at POD 1 was not significantly lower in the standard-care group (median (IQR), 28.6 (12.0–46.5) pg/mL) than in the NIRAF group (median (IQR), 33.2 (21.9–48.1) pg/mL).	The postoperative hypocalcemia rate was significantly lower in the NIRAF group (9.1% (95% CI, 4.0–14.2%)) than in the standard-care group (21.7% (95% CI, 14.3–29.0%); *p* = 0.007).
(Kose et al., 2020) [46]	For patients that underwent thyroidectomy, AF was demonstrated in 496 (98.6%) of the PGs; 33% had been first identified with NIRAF prior to visual recognition by the surgeon. In 5%, NIFI helped identify incidentally resected PGs.	Not measured	Not measured
(Enny et al., 2020) [47]	Two PGs in 30 (44.4%) patients, 0 PGs in 6 (6.9%) patients, and 4 PGs in 7 (9.7%) patients were visualized with fluorescein dye. With naked eye evaluation, 0 PGs in 1 patient, 2 PGs in 29 (41.7%) patients, and 4 PGs in 11 (15.3%) patients were visualized.	Not measured	Clinical hypocalcemia was observed in all patients in whom no PGs were visualized with fluorescein dye, whereas none of the patients in whom three or four PGs were visualized developed hypocalcemia. Among patients in whom three or four PGs were observed by the naked eye, 7 (28%) and 3 (23.7%) patients developed clinical hypocalcemia, respectively.
(Razavi et al., 2019) [48]	Not specified	Mean postoperative PTH decreased by 23.48 pg/mL for conventional care and 29.24 pg/mL for ICG.	Symptomatic hypocalcemia was observed in 3.90% of those who underwent conventional treatment and 7.90% of those in the ICG group.
(McWade et al., 2019) [43]	In total, 67 (97%) of exposed tissues of interest were correctly visualized as PGs.	Not measured	Not measured
(Rudin et al., 2019) [49]	Identification and autotransplantation were more common in the ICGA group at 36%, compared with 12% in the control group (*p* = 0.0001).	At POD 1, PTH was found to be low in 36% of controls and 37% of ICGA patients. An undetectable PTH level was present in 14% of control patients and 15% of ICGA patients. One patient in each group had permanent hypoparathyroidism.	Not measured
(van den Bos et al., 2019) [50]	In total, 41 PGs were visualized with white light in 25 patients, whereas 31 PGs were identified in 23 patients by NIRAF imaging.	Not measured	Three patients had transient hypocalcemia that resolved after 2 weeks.
(Falco et al., 2017) [51]	The mean number of identified PGs was 2.5 (±0.8) and 3.7 (±0.7) with WL (white light) and NIRAF respectively. In 86.5% (*n* = 64) of patients, four PGs were identified with NIFI, whereas four PGs were visualized with WL in only 12.2% (*n* = 9) of patients.	Not measured	Not stated
(Lang et al., 2017) [52]	A total of 340 PGs were identified, and 324 (95.3%) PGs were later confirmed to be PGs on histology.	Not measured	Nine (12.9%) patients developed transient hypocalcemia, while no patients had permanent hypocalcemia. There was a significant relationship between intensity of fluorescence image and the development of hypocalcemia. No patients with a greatest fluorescent light intensity developed postoperative hypocalcemia
(Serra et al., 2020) [53]	The mean number of PGs identified per patient was 3.47 in the study group and 2.33 in the control group (*p* < 0.0001).	Determinations of PTH 24 h after surgery showed a statistically significant difference favoring the study group.	In the study group, 24.4% presented 24-h postoperative hypocalcemia vs. 30% of the control group. At 6 months postoperation, three patients in the control group had permanent hypocalcemia, compared with no patients in the study group.
(Lerchenberger et al., 2019) [54]	A total of 64 (82%) PGs were visualized with AF; AF could not indicate whether the blood supply was still viable. On ICG administration, 63 PGs (81%) showed persistent fluorescence after a decrease in thyroid fluorescence.	Not measured	Only two patients developed transient hypocalcemia. No patients had permanent hypocalcemia
(De Leeuw et al., 2016) [55]	In total, 80 PGs were identified using the NIR system, and 81 glands were confirmed on frozen section to be PGs.	Not measured	Not measured
(Llorente et al., 2020) [56]	Not specified	Not measured	Eleven (22%) patients developed postoperative hypocalcemia. ICGA would allow immediate decision-making without the need to wait for intraoperative PTH measurements.
(S. W. Kim et al., 2016) [42]	All PGs were visualized.	No patient had postoperative hypoparathyroidism.	Not measured
(S. W. Kim et al., 2018) [57]	All but one PG were identified in vivo and preserved. The excised PG was autotransplanted	Not measured	Only one patient had transient hypocalcemia.
(R. Ladurner et al., 2018) [58]	Of the 41 PGs examined, 37 were identified by AF. AF assisted the preservation and autotransplantation of PGs in two patients	Not measured	Not measured
(Roland Ladurner et al., 2019) [59]	In total, 179 PGs (87.3%) displayed NIRAF showing a typical bluish violet color.	Not measured	Not measured
(Vidal Fortuny et al., 2016) [60]	Of the 36 patients who underwent ICGA, 30 had an ICG score of 2 for at least one PG. Autotransplantation was performed for those with poor ICG scores.	In the 30 patients with at least one PG with an ICG score of 2, postoperative PTH levels were in the normal range.	The postoperative adjusted calcium levels were within the normal range in 29 (80.6%) patients.
(Vidal Fortuny et al., 2018) [61]	In 146 patients, at least one preserved PG had an ICG score of 2.	Hypoparathyroidism was not observed in either group.	Hypocalcemia was not observed in either group.
(Dip et al., 2019) [62]	With NIRI, an average of 2.6 (0.85) PGs were detected prior to dissection. In four patients, PGs were transplanted after identification with NIRI.	Not measured	Significantly higher mean serum calcium levels were observed in the study group, with 8.2% having serum calcium <8 mg/dL compared with 16.5% in the control group. However, 1.2% in both groups required long-term calcium replacement, which was resolved by 6 months.
(Zaidi et al., 2016) [63]	A total of 71 (84%) PGs were identified on fluorescence.	The mean POD-1 PTH level of patients with at least two glands exhibiting <30% fluorescence at completion of thyroidectomy was 9 pg/dL, whereas those with fewer than two glands demonstrating <30% fluorescence had a POD-1 PTH of 19.5 pg/dL (*p* = 0.05).	Postoperatively, three patients (11%) had a serum calcium value <8 mg/dL, and one patient was symptomatic.
(Y. S. Kim et al., 2020) [64]	The mean number of PGs identified intraoperatively was similar between the two groups. The rate of incidental parathyroidectomy reported by pathology was higher in the conventional group (14%) than in the NIFI group (6%) (*p* = 0.039).	The POD-1 value was 23.9 pg/mL (17.6) in the NIRI group and 23.0 pg/mL (22.4) in the control group. The POD-14 level was 38.9 pg/mL (35.5) in the NIRI group and 35.8 pg/mL (26.1) in the control group.	At POD 1, the NIFI group had a level of 9.0 mg/dL (0.6) compared with 8.8 mg/dL (0.6) for the control group (*p* = 0.004). At POD 14, the NIFI group had a value of 8.8 mg/dL (0.6), compared with 8.6 mg/dL (0.6) for the control group (*p* = 0.008). All 5 patients with postoperative hypocalcemia in the NIFI group recovered within 2 weeks, while 1 of 14 patients with postoperative hypocalcemia in the conventional group had persistent hypocalcemia beyond 6 months.
Jin & Cui, 2020) [65]	In total, 186 PGs were visualized in 56 patients.	No patient in either group developed hypoparathyroidism.	No patient in either group developed hypocalcemia.
(D. H. Kim et al., 2021) [66]	PGs were found in 244 cases (93.5%) in the NIRAF group and in 260 cases (92.5%) in the control group. The mean count of identified PGs was 3.91 ± 0.36 in the NIRAF group vs. 3.90 ± 0.39 in the control group (*p* = 0.351).	The incidence of transient postoperative hypoparathyroidism was significantly lower in the NIRAF group than in the control group during hospitalization (33.7% vs. 46.6%; *p* = 0.002) and at 1 month (8.8% vs. 18.9%; *p* = 0.001)	The incidence of hypocalcemia during hospitalization was 6.5% in the NIRAF group and 10.0% in the control group. There was no significant difference in the rate of hypocalcemia between the two groups for any follow-up period.

PG—Parathyroid gland; ICG—Indocyanine green; NIRI—Near-infrared imaging; POD—postoperative day; AF—autofluorescence; NIRAF—near-infrared autofluorescence.

## Data Availability

No new data were created or analyzed in this study. Data sharing is not applicable to this article.
